# HIV prevalence in suspected Ebola cases during the 2014–2016 Ebola epidemic in Sierra Leone

**DOI:** 10.1186/s40249-019-0525-9

**Published:** 2019-03-04

**Authors:** William J. Liu, Hai-Yang Hu, Qiu-Dong Su, Zhe Zhang, Yang Liu, Yu-Lan Sun, Xian-Da Yang, Da-Peng Sun, Shao-Jian Cai, Xiu-Xu Yang, Idrissa Kamara, Abdul Kamara, Matt Lebby, Brima Kargbo, Patricia Ongpin, Xiao-Ping Dong, Yue-Long Shu, Wen-Bo Xu, Gui-Zhen Wu, Michael Gboun, George F. Gao

**Affiliations:** 1 NHC Key Laboratory of Biosafety, National Institute for Viral Disease Control and Prevention, Chinese Center for Disease Control and Prevention(China CDC), Beijing, 102206 China; 2 Jiangsu Provincial Center for Disease control and Prevention, Nanjing, 210009 China; 3 Beijing Institute of Biotechnology, Beijing, 100071 China; 4 Beijing Center for Disease Prevention and Control, Beijing, 100013 China; 5 Jilin Provincial Center for Disease control and Prevention, Changchun, 130021 China; 6 Shandong Provincial Center for Disease control and Prevention, Jinan, 250014 China; 7 Fujian Provincial Center for Disease control and Prevention, Fuzhou, 350001 China; 8 The Ministry of Health and Sanitation, Freetown, Sierra Leone; 9 UNAIDS, Freetown, Sierra Leone; 10 Chinese Center for Disease Control and Prevention, Beijing, 102206 China; 11 CAS Key Laboratory of Pathogenic Microbiology and Immunology, Institute of Microbiology, Chinese Academy of Sciences (CAS), Beijing, 100101 China; 12 Sierra Leone-China Friendship Biological Safety Laboratory, Freetown, Sierra Leone

**Keywords:** HIV, HCV, Ebola, Prevalence, Sierra Leone

## Abstract

**Background:**

The 2014–2016 Ebola virus epidemic in West Africa was the largest outbreak of Ebola virus disease (EVD) in history. Clarifying the influence of other prevalent diseases such as human immunodeficiency virus infection and acquired immune deficiency syndrome (HIV/AIDS) will help improve treatment and supportive care of patients with EVD.

**Case presentation:**

We examined HIV and hepatitis C virus (HCV) antibody prevalence among suspected EVD cases from the Sierra Leone-China Friendship Biological Safety Laboratory during the epidemic in Sierra Leone. HIV and HCV antibodies were tested in 678 EVD-negative samples by enzyme-linked immunosorbent assay. A high HIV prevalence (17.6%) and low HCV prevalence (0.22%) were observed among the suspected cases. Notably, we found decreased HIV positive rates among the suspected cases over the course of the epidemic. This suggests a potentially beneficial effect of an improved public health system after assistance from the World Health Organization and other international aid organizations.

**Conclusions:**

This EVD epidemic had a considerable impact on the public health system and influenced the prevalence of HIV found among suspected cases in Sierra Leone, but also provided an opportunity to establish a better surveillance network for infectious diseases.

**Electronic supplementary material:**

The online version of this article (10.1186/s40249-019-0525-9) contains supplementary material, which is available to authorized users.

## Multilingual Abstract

Please see Additional file 1 for translations of the abstract into the five official working languages of the United Nations.

## Background

The 2014–2016 Ebola virus (EBOV) epidemic in West Africa was the largest outbreak of Ebola virus disease (EVD) in history, causing over 28 616 infections and 11 310 deaths by June 2016 [1]. Sierra Leone, as one of the least developed countries in the world, was one of the three countries most seriously impacted during the outbreak. Clarifying the influence of other prevalent diseases such as HIV/AIDS will help improve treatment and supportive care of patients with EVD [2].

The Sierra Leone Demographic and Health Survey in 2008 and 2013 showed that 1.5% of Sierra Leonean adults aged 15–49 years were HIV-positive and that the HIV prevalence was slightly higher among women (1.7%) than men (1.3%) [3, 4]. However, some groups had reported that these rates were dramatically underestimated. From November 2014 to March 2015, suspected EVD-patients were admitted to the Sierra Leone-China Friendship Hospital in Sierra Leone and assessed for EBOV infection via real-time polymerase chain reaction (PCR) test of blood samples. Out of 278 EBOV-negative patients, 44 (15.83%) were diagnosed as HIV-positive [5]. During the same period at Moyamba Ebola Treatment Center (ETC), Sierra Leone, three out of 44 EBOV-negative patients (8.8%) were HIV-positive [6]. To further understand HIV prevalence among suspected Ebola cases in Sierra Leone, we tested blood samples for the presence of HIV antibodies among suspected EVD patients at a biosafety level-3 laboratory, examined the prevalence of HIV, and analysed the probable correlation between HIV and EVD epidemic in Sierra Leone.

## Case presentation

From March to November 2015, the Sierra Leone-China Friendship Biological Safety Laboratory received a total of 901 blood samples collected from 731 EVD-suspected patients. Of these samples, 94 from 53 individuals were EBOV positive as confirmed by real-time PCR, while 807 samples from 678 individuals tested EBOV negative by real-time PCR. The 678 EBOV-negative samples were first tested for HIV antibodies using an enzyme-linked immunosorbent assay (ELISA; Beijing Wantai Biological Pharmacy Enterprise Co., Ltd., Beijing, China) and then retested using another ELISA reagent (Zhuhai Livzon Diagnostics Inc., Zhuhai, China). HIV positivity was defined as having positive results in the two tests [7]. The EVD-negative samples were also tested for hepatitis C virus (HCV) antibodies via ELISA (Beijing Wantai Biological Pharmacy Enterprise Co., Ltd., Beijing, China).

All data were entered into a Microsoft Excel spreadsheet (2016, Microsoft, Redmond, USA). All data analyses were performed using SPSS version 19.0 (SPSS Inc., Chicago, IL, USA). Frequency analyses were used to calculate the HIV-antibody and HCV-antibody positivity rates among all EBOV-negative patients and subgroups. Chi-square tests were used to compare differences between subgroups. Chi-square trend tests were used to evaluate the trend of HIV prevalence. Pearson’s correlation coefficient analyses were used to evaluate the correlation between the monthly HIV prevalence and reported Ebola cases. Differences with *P*-values less than 0.05 were considered statistically significant.

Of the 678 EBOV-negative patients, 55.7% (372/668) were male, while 44.3% (296/668) were female (Table 1). Regarding age distribution, the 0–14 year age group constituted 24.7% (162/656), the 15–49 year age group constituted 63.1% (414/656), and the 50-year-old or older age group constituted 12.2% (80/656) of the entire cohort. Regarding geographical distribution, 64.9% (440/678) was from the Western Urban area, 29.8% from the Western Rural area, and 5.3% from other or unknown areas.

**Table 1:** The distributions of HIV prevalence by gender, age and district (*n*=678)

Variable	Negative (n)	Positive (n)	Prevalence (%)	χ^2^	*P*-value
Gender				23.972	<0.001
Female	220	76	25.7		
Male	330	42	11.3		
unknown	9	1	10		
Age group (years)				48.511	<0.001
0-14	158	4	2.5		
15-49	312	102	24.6		
50	74	6	7.5		
unknown	15	7	31.8		
District				6.695	0.035
Western Urban	375	65	14.8		
Western Rural	156	46	22.8		
Other and unknown	28	8	22.2		

Among EBOV-negative patients, the mean HIV-antibody positivity rate was 17.6%, with 11.3% in males, 25.7% in females, 2.5% in 0–14 age group, 24.6% in 15–49 age group, 7.5% in 50 years old or older group, 14.8% in Western Urban group and 22.8% in Western Rural group (Table 1). In our study, the distribution features of HIV prevalence by gender and age were consistent with the previous reports in Sierra Leone and other African countries [8]. This may be related to the different characteristics of multiple sexual partners and condom usage in populations with different gender and age distributions. For the HCV antibody test, only one positive sample was detected, indicating a low HCV prevalence (0.22%).

**Fig. 1:**
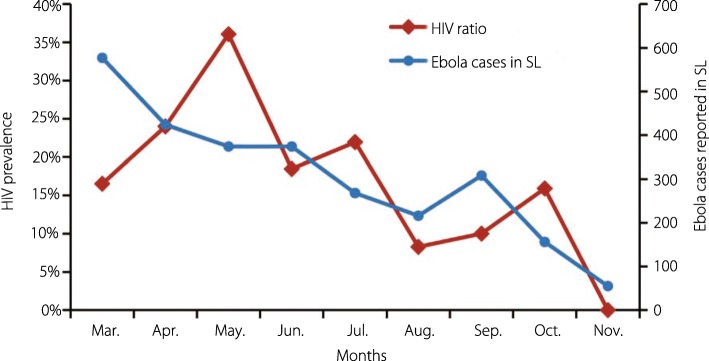
The prevalence of HIV among EBOV-negative EVD-suspected cases tested at Sierra Leone-China Friendship Biological Safety Laboratory, Sierra Leone, 2015. After its establishment in early 2015, the Sierra Leone-China Friendship Biological Safety Laboratory was responsible for EBOV testing of EVD-suspected patients from most districts of the country. From March to November 2015, 678 individuals were excluded from EBOV infection by real-time PCR method. HIV antibodies in the sera were tested using ELISA (with kits from Beijing Wantai Biological Pharmacy Enterprise Co., Ltd., Beijing, China, and positive cases were confirmed using kits from Zhuhai Livzon Diagnostics Inc., Zhuhai, China). The significant decreases (*P* < 0.001 in Chi-square tests) in HIV prevalence ratio among the Ebola RNA negative suspected Ebola cases in each month are shown as brown lines and diamond symbols. The monthly reported number of Ebola cases in Sierra Leone by the WHO are shown as blue lines and diamond symbols (http://apps.who.int/gho/data/view.ebola-sitrep.ebola-summary-latest?lang=en) and were significantly correlated with the reported monthly number of Ebola cases (*r* = 0.745, *P* = 0.034) using Pearson’s correlation coefficient analyses. EBOV: Ebola virus; EVD: Ebola virus disease; PCR: Polymerase chain reaction; ELISA: Enzyme-linked immunosorbent assay

We then evaluated the HIV positivity rate among the suspected EVD cases. From March to July 2015, the prevalence of HIV infection among the suspected EVD cases was 22.5% and over 16% per month, including up to 36% in May. From August to November 2015, the prevalence was 9.8% and less than 16% per month, including zero in November. From April to November 2015, the prevalence of HIV infection among the suspected EVD cases showed a significant decrease (*P* < 0.001) in Chi-square value and a significant correlation with the monthly-reported number of EVD cases (*r* = 0.745, *P* = 0.034) by Pearson’s correlation coefficient analyses (Fig. 1).

## Discussion and conclusions

The 2014–2016 EVD outbreak in West Africa resulted in the disruption of an already fragile but essential health service and drug distribution system. Particularly, HIV clinical services in affected countries were also affected [9]. The death of several healthcare workers in early 2014, as well as the strain on healthcare facilities caused by increased numbers of EVD patients and decreased numbers of staff, resulted in the closure of many clinics and the interruption of routine health delivery services, including HIV testing, antiretroviral therapy (ART) [10], prevention of mother-to-child transmission of HIV [11], childhood vaccination, and maternity care. This may impact on other infectious diseases, such as AIDS, malaria, tuberculosis, and measles [12, 13]. A recent computational simulation model has estimated a 50% reduction in ART coverage and 10% increase in AIDS-related deaths attributable to this EVD outbreak in Sierra Leone from March 2014 to March 2015 [14]. Thus, HIV/AIDS patients may have been inclined to visit the ETCs for medical care during the EVD outbreak.

From January to July 2015, the EVD outbreak entered the second phase of the response, during which great efforts were undertaken by the World Health Organization (WHO) and partners, including enhancing capacities for case finding, increasing efforts for contact tracing, and community engagement. Strengthened surveillance and response systems resulted in many suspected EVD patients suffering from other diseases visiting ETCs. Thus, many suspected EVD cases were indeed HIV/AIDS-positive as they exhibited many of the same symptoms and/or because many AIDS patients had defaulted their treatments. During the second half of 2015, with the help of the WHO and other international assistances including the support from Chinese Center for Disease Control and Prevention, the overarching goal of interrupting the remaining chains of EBOV transmission was completed, and the public health system was being re-established. Moreover, the public health situations in Sierra Leone had improved to respond to the surveillance of several other dominant infectious diseases, including malaria and HIV/AIDS. Thus, HIV-infected persons may now visit the HIV/AIDS service units again. Before admission to ETCs, HIV-infected persons were differentiated from suspected EVD cases using specific tests. Accordingly, this may have led to a decreased HIV-positive ratio among EVD suspected cases.

In conclusion, our study indicates an interaction between the EVD outbreak and other important infectious diseases prevalent in the same area. The 2014–2016 EVD epidemic had a considerable impact on the public health system and influenced the prevalence of HIV found among suspected EVD cases in Sierra Leone. However, it also accelerates the establishment of a better surveillance network for infectious diseases. During the post-EBOV era, a passive surveillance system was insufficient for identifying more cases of HIV infection. Other innovative and effective strategies should be implemented to expand HIV testing in Sierra Leone. A well-established disease control and prevention system in Africa will make invaluable contributions to global health.

## Additional file

Additional file 1: Multilingual abstracts in the five official working languages of the United Nations. (PDF 230 kb)

## Abbreviations

ART: Antiretroviral therapy
EBOV: Ebola virus
ELISA: Enzyme-linked immunosorbent assay
ETC: Ebola treatment center
EVD: Ebola virus disease
HCV: Hepatitis C virus
PCR: Polymerase chain reaction
WHO: World Health Organization HIV/AIDS Human immunodeficiency virus infection and acquired immune deficiency syndrome
