# Supplementary material for: HIV prevalence in suspected Ebola cases during the 2014–2016 Ebola epidemic in Sierra Leone
Source: Infect Dis Poverty. 2019 Mar 4;8:15. doi: 10.1186/s40249-019-0525-9 (PMC6398227; doi:10.1186/s40249-019-0525-9)
Supplement: Supplementary file 1 — Multilingual abstracts in the five official working languages of the United Nations. (PDF 230 kb) [file 40249_2019_525_MOESM1_ESM.pdf]

Translation of the abstract into the five official working languages of the United Nations

انتشار فيروس نقص المناعة البشرية في حالات الإيبولا المشتبه فيها خلال وباء الإيبولا 2014-2014 في سيراليون

وليام ج. ليو، هاي-يانج ها، كيو-دونج سو، شي زانج، يانج ليو، ي-لان سون، كسيان-دا يانج، دا-بنج سان، شاو-جيان كي، كسيو-كسوبريما كركامارا، مات لبي، يانج، إدريسا كامارا، عبدول كامارا، مات لبي، بريما كارجبو، باتريسيا أونجبين، كسياو-بينج دونج، يو-لونج شو، وين-بو كسو، جي-زان وو مايكل جبون، جورج ف. جاو

ملخص

الخلفية: يعتبر انتشار وباء فيروس الإيبولا ما بين 2014-2016 في غرب إفريقيا الأكثر تفشيا لمرض فيروس الإيبولا (EVD) في التاريخ. إن من شأن توضيح تأثير الأمراض السارية الأخرى مثل عدوى فيروس العوز المناعي البشري ومتلازمة نقص المناعة المكتسبة (HIV / AIDS) أن يساعد على تحسين العلاج والرعاية الداعمة للمرضى الذين يعانون من فيروس إيبولا(EVD).

عرض الحالة: فحصنا فيروس نقص المناعة وفيروس التهاب الكبد الوبائيانتشار الأجسام المضادة بين الحالات المشتبه بإصابتها بفيروس الإيبولا لدى مختبر السلامة البيولوجية للصداقة بين سيراليون والصين أثناء انتشار الوباء في سيراليون. تم اختبار الأجسام المضادة لفيروس نقص المناعة البشرية وفيروس التهاب الكبد الفيروسي على 678 عينة سلبية غير مصابة بفيروس إيبولا وذلك عن طريق مقايسة الامتصاص المناعي المرتبط بالإنزيم. لوحظ ارتفاع معدل انتشار فيروس نقص المناعة البشرية (17.6 ٪) وانخفاض انتشار فيروس الإلتهاب الكبدي (0.22 ٪) بين الحالات المشتبه فيها. ومن الجدير بالملاحظة أننا وجدنا انخفاضا بمعدلات الإصابة بفيروس نقص المناعة البشرية بين الحالات المشتبه فيها على مدار انتشار الوباء. وهذا يشير إلى التأثير المفيد المحتمل لنظام تحسين الصحة العامة بعد مساعدة منظمة الصحة العالمية ومنظمات المعونة الدولية الأخرى.

الاستنتاجات: لقد كان لهذا الوباء تأثير كبير على نظام الصحة العامة وأثر على انتشار فيروس نقص المناعة البشرية بين حالات مشتبه بها في سيراليون، بالإضافة إلى إتاحة الفرصة لإنشاء شبكة مراقبة أفضل للأمراض المعدية.

Translated from English version into Arabic by Malika El Khadhri and by Amro Salah, through

TRANSLATORS
WITHOUT BORDERS

2014-2016 年塞拉利昂疑似埃博拉病人中艾滋病病毒的感染率

William J. Liu, Hai-Yang Hu, Qiu-Dong Su, Zhe Zhang, Yang Liu, Yu-Lan Sun, Xian-Da Yang, Da-Peng Sun, Shao-Jian Cai, Xiu-Xu Yang, Idrissa Kamara, Abdul Kamara, Matt Lebby, Brima Kargbo, Patricia Ongpin, Xiao-Ping Dong, Yue-Long Shu, Wen-Bo Xu, Gui-Zhen Wu, Michael Gboun, George F. Gao

摘要

引言：2014-2016 年西非埃博拉的暴发流行是埃博拉病毒病流行有史以来最大的一次，同时这也对其他传染病例如艾滋病的流行与防控产生很大影响。

方法与结果：针对塞拉利昂-中国友好生物安全实验室在埃博拉流行期收到并检测的疑似埃博拉病人的血样，本研究对这些样本进行 HIV 抗体及 HCV 抗体的检测以获得在这一人群的流行状况。应用酶联免疫吸附试验对 678 份埃博拉阴性血样进行 HIV 抗体及 HCV 抗体的检测。HIV 抗体阳性率为 17.6%，HCV 抗体阳性率为 0.22%，其中 HIV 的数据高于既往的监测数据。特别是研究发现疑似埃博拉病人 HIV 阳性率随着埃博拉疫情逐渐平息而降低，这体现了在 WHO 和其他国际组织的帮助下，重建的公共卫生体系已发挥的作用。

结论：这次的埃博拉流行对西非国家公共卫生体系产生了巨大的影响，包括对艾滋病防控的影响。然而，这同样也提供了一个机会来建立一个更好的传染病监测网络。

Translated from English version into Chinese by William J. Liu

Prévalence du VIH parmi les cas suspects de fièvre d'Ebola au cours de l'épidémie de virus Ebola de 2014-2016 au Sierra Leone

William J. Liu, Hai-Yang Hu, Qiu-Dong Su, Zhe Zhang, Yang Liu, Yu-Lan Sun, Xian-Da Yang, Da-Peng Sun, Shao-Jian Cai, Xiu-Xu Yang, Idrissa Kamara, Abdul Kamara, Matt Lebby, Brima Kargbo, Patricia Ongpin, Xiao-Ping Dong, Yue-Long Shu, Wen-Bo Xu, Gui-Zhen Wu, Michael Gboun, George F. Gao

Résumé

Contexte : l'Afrique de l'Ouest a connu la plus grande et la plus longue épidémie de fièvre d'Ebola entre 2014 et 2016. Connaître plus précisément l'influence d'autres maladies prévalentes, comme l'infection par le virus de l'immunodéficience humaine (VIH) et le syndrome d'immunodéficience acquise (SIDA), aiderait à améliorer le traitement et les soins palliatifs des patients atteints par la maladie à virus Ebola.

Présentation de cas : nous avons examiné la prévalence du VIH et du virus de l'hépatite C (VHC) parmi des cas suspectés d'infection par le virus Ebola au Laboratoire de sécurité biologique de l'Amitié Sierra-Leone Chine pendant l'épidémie au Sierra Leone. Les anticorps anti-VIH et anti-VHC ont été recherchés par immuno-absorption enzymatique dans 678 échantillons négatifs pour le virus Ebola. Une forte prévalence du VIH (17,6 %) et une faible prévalence du VHC (0,22 %) ont été observées parmi les cas suspects. Nous avons notamment constaté une baisse du taux de résultats positifs pour le VIH parmi les cas suspects au cours de l'épidémie. Cela suggère un effet potentiellement bénéfique des améliorations apportées au système de santé publique avec l'aide de l'OMS et d'autres organismes d'aide internationaux.

Conclusions : l'épidémie de virus Ebola a eu un impact considérable sur le système de santé publique et a influencé la prévalence des cas positifs de VIH retrouvés parmi les cas suspects au Sierra Leone. Elle a cependant aussi donné l'occasion de mettre en place un meilleur réseau de surveillance des maladies infectieuses.

Translated from English version into French by Suzanne Assenat and Eric Ragu, through

Распространенность ВИЧ-инфекции среди лиц с подозрением на лихорадку Эбола во время эпидемии Эболы в Сьерра-Леоне в 2014–2016 гг.

Уильям Дж. Лю, Хай-Ян Ху, Цю-Донг Су, Чжэ Чжан, Ян Лю, Ю-Лан Сунь, Сиань-Да Ян, Да-Пэн Сунь, Шао-Цзянь Цай, Сю-Сюй Ян, Идрисса Камара, Абдул Камара, Мэтт Лебби, Брима Каргбо, Патриция Онгпин, Сяо-Пинг Донг, Юэ-Лонг Шу, Вэнь-Бо Сюй, Гуй-Чжэнь Ву, Майкл Гбоун, Джордж Ф. Гао

Аннотация

Справка: Эпидемия вируса Эбола в Западной Африке в 2014–2016 годах была самой крупной в истории вспышкой геморрагической лихорадки Эбола (EVD). Разъяснение влияния других распространенных заболеваний, таких как вирус иммунодефицита человека и синдром приобретенного иммунодефицита (ВИЧ/СПИД), поможет улучшить лечение и поддержку пациентов с EVD.

Исследование: Мы исследовали распространенность антител ВИЧ и вируса гепатита С (HCV) среди случаев подозрения на геморрагическую лихорадку Эбола из Лаборатории биологической безопасности дружбы Сьерра-Леоне и Китая во время эпидемии в Сьерра Леоне. Антитела к ВИЧ и вирусу гепатита С были протестированы на 678 Эбола-негативных образцах с помощью иммуноферментного анализа. Среди подозреваемых случаев была отмечена высокая распространенность ВИЧ (17,6%) и низкая распространенность вирусного гепатита С (0,22%). Примечательно, что в ходе эпидемии мы обнаружили снижение числа ВИЧ-позитивных среди подозреваемых случаев. Это предполагает потенциально полезный эффект от улучшения системы общественного здравоохранения после помощи Всемирной организации здравоохранения и других международных организаций.

Заключение: Эта эпидемия лихорадки Эбола оказала значительное влияние на систему общественного здравоохранения и повлияла на распространенность ВИЧ-инфекции среди подозреваемых случаев в Сьерра-Леоне, но также предоставила возможность создать лучшую сеть эпиднадзора за инфекционными заболеваниями.

Translated from English version into Russian by Tatiana Karymshakova and Michael Orlov, through

Prevalencia del VIH en los presuntos casos de Ébola durante la epidemia del Ébola del 2014

al 2016 en Sierra Leona

William J. Liu, Hai-Yang Hu, Qiu-Dong Su, Zhe Zhang, Yang Liu, Yu-Lan Sun, Xian-Da Yang, Da-Peng Sun, Shao-Jian Cai, Xiu-Xu Yang, Idrissa Kamara, Abdul Kamara, Matt Lebby, Brima Kargbo, Patricia Ongpin, Xiao-Ping Dong, Yue-Long Shu, Wen-Bo Xu, Gui-Zhen Wu, Michael Gboun, George F. Gao

Resumen

Antecedentes: La epidemia del virus del Ébola del 2014 al 2016 en África Occidental fue el mayor brote de la enfermedad del virus del Ébola (EVE) de la historia. Explicar la influencia de otras enfermedades frecuentes, como la infección por el virus de la inmunodeficiencia humana y el síndrome de inmunodeficiencia adquirida (VIH/SIDA), ayudará a mejorar el tratamiento y los cuidados de paliativos de los pacientes con EVE.
Presentación del caso: Se examina la prevalencia de anticuerpos del VIH y del virus de la hepatitis C(VHC) entre los presuntos casos de EVE del Laboratorio de Seguridad Biológica de la Amistad de Sierra Leona-China durante la epidemia ocurrida en Sierra Leona. Los anticuerpos del VIH y del VHC se analizaron en 678 muestras EVE negativas mediante la prueba de inmunoabsorción enzimática. Se observó una alta prevalencia del VIH (17,6%) y una baja prevalencia del VHC (0,22%) entre los presuntos casos. En particular, encontramos una disminución de las tasas positivas del VIH entre los presuntos casos en el transcurso de la epidemia. Esto sugiere un efecto potencialmente beneficioso de un sistema de salud pública que ha mejorado después del apoyo ofrecido por la Organización Mundial de la Salud y de otras organizaciones de ayuda internacional.
Conclusiones: Esta epidemia de EVE tuvo un impacto considerable en el sistema de salud pública e influyó en la prevalencia del VIH detectado entre los presuntos casos en Sierra Leona; sin embargo, también proporcionó la oportunidad de establecer y mejorar la red de vigilancia de las enfermedades infecciosas.

Translated from English version into Spanish by Lia Sarra Felip and María Isabel Barranquero, through

TRANSLATORS
WITHOUT BORDERS
